# Influence of body mass and environmental conditions on winter mortality risk of a northern ungulate: Evidence for a late‐winter survival bottleneck

**DOI:** 10.1002/ece3.6026

**Published:** 2020-01-21

**Authors:** Todd M. Kautz, Jerrold L. Belant, Dean E. Beyer, Bronson K. Strickland, Jared F. Duquette

**Affiliations:** ^1^ Camp Fire Program in Wildlife Conservation State University of New York College of Environmental Science and Forestry Syracuse NY USA; ^2^ Wildlife Division Michigan Department of Natural Resources Marquette MI USA; ^3^ Forest and Wildlife Research Center Mississippi State University Mississippi State MS USA; ^4^ Illinois Department of Natural Resources Champaign IL USA

**Keywords:** *Canis latrans*, *Canis lupus*, cause‐specific mortality, *Odocoileus virginianus*, weather, winter severity index

## Abstract

1. A relationship between winter weather and survival of northern ungulates has long been established, yet the possible roles of biological (e.g., nutritional status) and environmental (e.g., weather) conditions make it important to determine which potential limiting factors are most influential.
2. Our objective was to examine the potential effects of individual (body mass and age) and extrinsic (winter severity and snowmelt conditions) factors on the magnitude and timing of mortality for adult (>2.5 years old) female white‐tailed deer (*Odocoileus virginianus* [Zimmerman, 1780]) during February–May in the Upper Peninsula of Michigan, USA.
3. One hundred and fifty deer were captured and monitored during 2009–2015 in two areas with varying snowfall. February–May survival ranged from 0.24 to 0.89 (mean = 0.69) across years. Mortality risk increased 1.9% with each unit increase in cumulative winter severity index, decreased 8.2% with each cumulative snow‐free day, and decreased 4.3% with each kg increase in body mass. Age and weekly snow depth did not influence weekly deer survival. Predation, primarily from coyote (*Canis latrans* [Say, 1823]) and wolves (*Canis lupus* [L., 1758]), accounted for 78% of known‐cause mortalities.
4. Our results suggest that cumulative winter severity, and possibly to a lesser degree deer condition entering winter, impacted deer winter survival. However, the timing of spring snowmelt appeared to be the most influential factor determining late‐winter mortality of deer in our study. This supports the hypothesis that nutrition and energetic demands from weather conditions are both important to northern ungulate winter ecology. Under this model, a delay of several weeks in the timing of spring snowmelt could exert a large influence on deer survival, resulting in a survival bottleneck.

## Graphical abstract

Northern ungulate population dynamics are correlated with winter weather patterns, and our goal was to evaluate when and why white‐tailed deer winter mortality occurs. We modeled weekly mortality risk of 150 adult female white‐tailed deer in Michigan in response to environmental and biological factors. The results suggested that body mass, severity of winter weather, and timing of spring snowmelt are influential on deer mortality, with the timing of snowmelt explaining the greatest amount of variation.

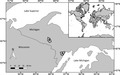

## 1 INTRODUCTION

Identifying limiting factors for animals with seasonally dynamic life histories sometimes requires understanding intra‐annual periods of resource scarcity (e.g., Ashmole, 1963). For ungulates living in northern environments, winter is generally a period of negative energy budget when forage provides some energetic intake but most individuals rely heavily on fat stores accumulated during the previous summer and fall to survive until spring green‐up (hereafter the nutritional integration model; Mautz, 1978; Parker, Barboza, & Gillingham, 2009). Following this model, an annual survival bottleneck around the time of snowmelt could occur if the intensity and duration of winter are sufficient to exceed the energetic reserves of a substantial portion of the population (Parker et al., 2009).

An important prediction of the nutritional integration model is that the magnitude of late‐winter survival bottlenecks is influenced by multiple mechanisms: Winter severity (e.g., depth of snow and temperature) determines rate of energetic expenditure, duration of snow cover determines how long a negative energy budget persists, and body fat reserves carried into the winter from previous foraging seasons determine how much energy is available to lose before succumbing to mortality from starvation or other causes affecting weakened animals (Parker et al., 2009). The importance of late‐winter survival for population dynamics of northern ungulates has been recognized (e.g., Clutton‐Brock, Price, Albon, & Jewell, 1991; Metz, Smith, Vucetich, Stahler, & Peterson, 2012), yet determining which individual or environmental factors limit wild ungulate populations during late winter remains difficult due to the possible interacting roles of biological (e.g., nutritional status) and environmental (e.g., weather) conditions (Wang et al., 2009).

In most large ungulate species, adult female survival is typically higher, more stable, and less sensitive to environmental change than juvenile or adult male survival (Gaillard & Yoccoz, 2003; McCullough, 1979). In North America and Europe, female ungulates can have a life span exceeding 15 years (Loison, Festa‐Bianchet, Gaillard, Jorgenson, & Jullien, 1999), but generally succumb to one of the numerous mortality agents (e.g., predation, starvation, disease, injury, exposure) before reaching their maximum potential longevity (Delgiudice, Fieberg, Riggs, Carstenson Powell, & Pan, 2006; Ericsson & Wallin, 2001). The magnitude and timing of mortality for adult female free‐ranging ungulates in temperate regions is influenced by habitat, predators, and weather with greatest nonhunting mortality often occurring during winter (Gaillard, Festa‐Bianchet, & Yoccoz, 1998; Forrester & Wittmer, 2012). Consequently, identifying which conditions result in high mortality risk for ungulates during winter is key to understanding what mechanisms are potentially limiting population growth.

**Figure 1:**
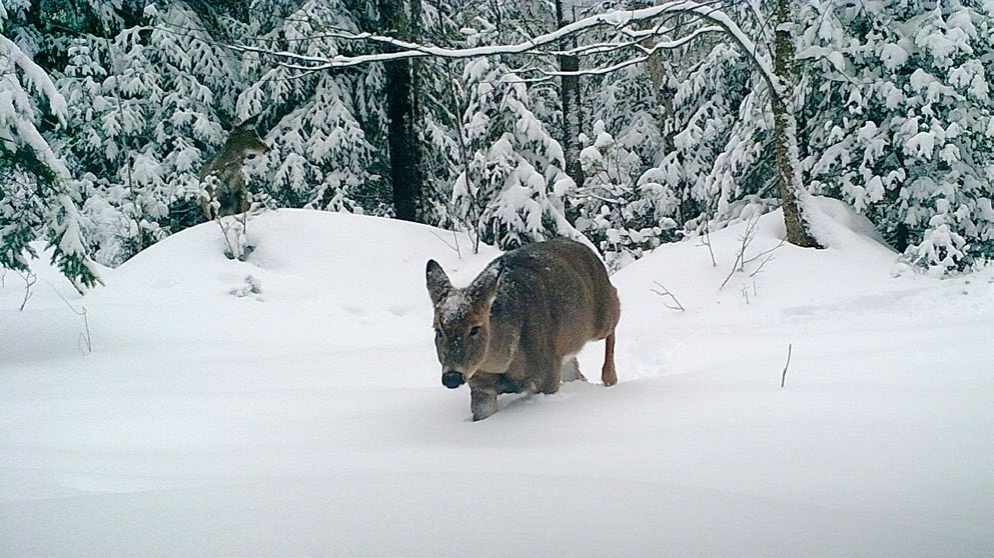
White‐tailed deer moving through snow during late winter in the Upper Peninsula of Michigan, USA

For white‐tailed deer (*Odocoileus virginianus* [Zimmerman 1780]; Figure 1; hereafter deer) populations near the northern edge of the species' range, late winter is a period of resource scarcity characterized by a negative energy budget, low fat reserves, and highly concentrated deer densities within suitable winter habitat (DelGiudice, Mech, Kunkel, Gese, & Seal, 1992; Dumont, Ouellet, Crête, & Huot, 2005; Mautz, 1978; Nelson, 1995). Consequently, adult mortality from predation and malnutrition in northern deer populations is generally greatest during winter, particularly during March–April (DelGiudice, Riggs, Joly, & Pan, 2002; DePerno, Jenks, Griffin, & Rice, 2000; Dumont, Crête, Ouellet, Huot, & Lamoureux, 2000; Van Deelen, Campa, Haufler, & Thompson, 1997; Whitlaw et al., 1998).

Determining which winter weather components relate most strongly to deer survival is an important topic for deer managers. The relationship between winter weather and survival of northern deer has led many natural resource agencies to adopt annual winter severity indexes (WSI) to predict cervid population trends (Verme, 1968; Leckenby & Adams, 1986; Chadwick, 2002; Delgiudice et al., 2002; Duquette et al., 2014). Generally, snow depth and temperature have been considered important predictors of deer mortality, with wind sometimes playing an important role in more open habitats. In addition, spring snowmelt timing may be important when considering winter severity for deer. Ignoring the middle period of winter and considering only the early and late months of winter may result in a better index of weather effects on deer (Verme, 1977), and spring snow depths can influence aspects of northern white‐tailed deer ecology including migration behavior (Nelson, 1995), habitat selection (Beier & McCullough, 1990), and natal mortality (Verme, 1977).

The Upper Peninsula of Michigan, USA, has a geographic gradient of snow conditions that is highly variable among years due to the climatic influence of the Great Lakes. Historically, deer population growth in the Upper Peninsula has been linked to variation in winter weather temporally and geographically (Doepker, Beyer, & Donovan, 1995; Leopold, Sowls, & Spencer, 1947). The Upper Peninsula deer population declined due to consecutive severe winters in 1995–1996 and 1996–1997 but did not fully recover over the next 15 years while the recolonizing gray wolf (*Canis lupus* [L. 1758]) population increased during this same period (Michigan Department of Natural Resources [MDNR], 2010, 2015). The adverse nutritional effects of winter in Michigan can result in substantial deer kill even in the absence of predators (Case & McCullough, 1987), but certain winter weather conditions may facilitate wolf predation of ungulates through either limiting prey mobility (e.g., deep or crusted snow) or by causing nutritional degradation that weakens prey (Mech & Peterson, 2003; Mech, Smith, Murphy, & MacNulty, 2001; Telfer & Kelsall, 1984; Vucetich et al., 2012). These events resulted in uncertainty of how deer in the Upper Peninsula are influenced by winter conditions in the presence of predators, and whether winter kill limits deer population growth.

Our goal was to test whether patterns of deer survival within late winter follow predictions from the nutritional integration model and determine which mechanisms most strongly influence survival. Cause‐specific mortality of adult female white‐tailed deer was investigated in relation to deer age, body mass, age, snow depth, cumulative effects of winter weather (described in Methods section), and cumulative effects of snow‐free days in two areas with differing amounts of snowfall. We based our predictions on the hypothesis that deer generally maintain a negative energy balance during winter at northern latitudes, that weather conditions shape the rate of this nutritional decline, and that survival is dependent on conserving energetic stores until spring snowmelt. We predicted that deer mortality risk would increase with greater snow depth, fewer snow‐free days during February–May, older and younger (nonprime) age classes, and decreasing body mass.

## 2 MATERIALS AND METHODS

### 2.1 Study sites

**Figure 2:**
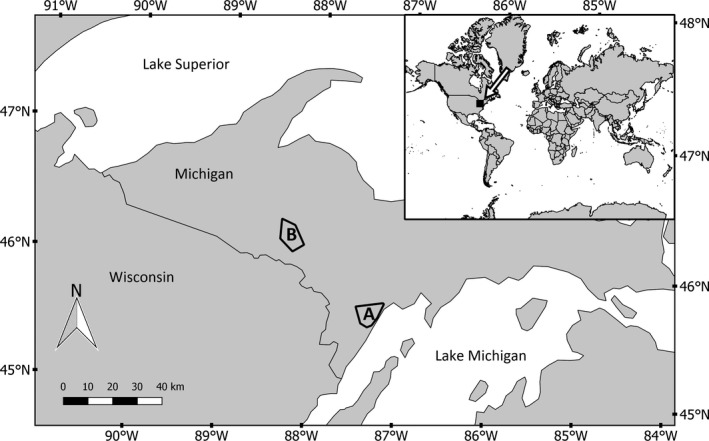
Location of low‐snowfall (A) and mid‐snowfall (B) study areas within the Upper Peninsula of Michigan, USA, 2009–2015

Data were collected from two study areas in the Upper Peninsula of Michigan, hereafter referred to as the low‐snowfall and mid‐snowfall study areas. Both study areas contained populations of gray wolf, coyote (*Canis latrans* [Say 1823]), and bobcat (*Lynx rufus* [Schreber 1777]). The low‐snowfall study area encompassed 319 km^2^ in Menominee County (45°24′00′′N 87°30′00′′W; Figure 2). Mean annual precipitation was 72.5 cm of rain and 128.8 cm of snow (1971–1996 averages, Michigan Climatology Office, 2013a). Mean January and July temperatures were –8°C and 19°C, respectively (PRISM Climate Group, 2016). Dominant land covers included woody wetlands (52%), deciduous forest (14%), and agricultural area (14%). The remaining 20% consisted of conifer forest, mixed forest, developed areas, herbaceous wetlands, shrub, and open water (Fry et al., 2011).

The mid‐snowfall study area included 341 km^2^ near the Michigamme Reservoir (46°14′00′′N 88°13′00′′W; Figure 2) and was 65 km northwest of the low‐snowfall study area. Mean annual precipitation was 52 cm of rain and 179 cm of snow (1951–1980 averages, Michigan Climatology Office, 2013b). Mean January and July temperatures were –13°C and 18°C, respectively (PRISM Climate Group, 2016). Land cover was predominantly deciduous forest (38%), woody wetland (29%), mixed forest (13%), and evergreen forest (6%) (Fry et al., 2011).

### 2.2 Deer capture and handling

Adult female white‐tailed deer were captured during February–April, 2009–2011, in the low‐snowfall study area and February–March, 2013–2015 in the mid‐snowfall study area. We captured deer primarily using Clover traps (Clover, 1956) baited with shelled corn, alfalfa, and/or molasses, and occasionally used cannon nets. Deer were restrained, blindfolded, and immobilized with an intramuscular injection of ketamine hydrochloride (Putney, Inc., Portland, ME, USA) and xylazine hydrochloride (Lloyd Laboratories, Shenandoah, IA, USA) mixed at a 4:1 ratio and concentration of 100 mg/ml (Duquette, Belant, Beyer, & Svoboda, 2013). For each deer, body mass was recorded and age estimated by extracting a lower incisiform canine to age deer using counts of cementum annuli (Gilbert, 1966; see Nelson[, 2002] for summary of ethics and effects of tooth removal) at the MDNR Diagnostic Center for Population and Animal Health (Lansing, MI, USA). We examined deer for pregnancy (enlarged caruncles, expanded uterus, and fetuses) using ultrasonography and attached transmitters if signs of pregnancy were observed. Each deer determined to be pregnant was fitted with a VHF collar with an 8‐hr movement mortality switch (Model M2510B; Advanced Telemetry Systems, Isanti, Minnesota, USA), and a vaginal implant transmitter with temperature switch and precise event transmitter to record time of temperature drop at half‐hour intervals for up to 128 hr (Model M3930; Advanced Telemetry Systems, Isanti, Minnesota, USA). Before release, each deer received an intravenous or intramuscular injection of yohimbine hydrochloride (ZooPharm, Laramie, WY, USA) to reverse the effects of xylazine hydrochloride (Duquette et al., 2013; Kreeger, Arnemo, & Raath, 2002). All animal handling procedures were approved by the Institutional Animal Care and Use Committee of Mississippi State University, Mississippi State, MS, USA (protocol number 12–012).

Deer were monitored on a weekly basis using aerial‐ or ground‐based telemetry. When a mortality signal was detected, the date and cause of mortality was determined based on deer remains and sign found at the mortality site. For predation events, evidence at the site (e.g., tracks, scat, canine puncture wounds, and site disturbance) was compared to published reports of predator‐specific kills to estimate predator species (Mech, Frenzel, & Karns, 1971; Nelson & Mech, 1986). Mortalities were classified as unidentified predations if the mortality site showed evidence of predation (e.g., blood in surrounding snow, hemorrhaging on hide or tissue), but evidence was insufficient to assign a predator species or evidence of multiple predator species was present. Malnutrition status of mortalities was assessed using rump fat and bone marrow condition (Mech, 2008) or by submitting carcasses for laboratory necropsy by a wildlife pathologist. In 68% of mortalities, investigations occurred <5.3 days after the time of mortality and date of mortality was determined to the nearest half hour using the precise event transmitter code of vaginal implant transmitters. For the remaining 32% of mortality events in which >5.3 days had passed, date of mortality was estimated using a combination of carcass decomposition, snow cover conditions, and telemetry records.

### 2.3 Weather data and deer density estimates

The area of data collection for weather variables was determined by calculating the minimum convex polygon of mid‐March aerial telemetry locations of deer, composite for all years within each study area. Ninety‐two percent of deer telemetry locations collected during periods of snow cover between November and May occurred within these polygons. Daily snow depth estimates from 1 November to 31 May for each winter were obtained using 0.4‐km resolution data from the National Snow and Ice Data Center Snow Data Assimilation System (National Operational Hydrologic Remote Sensing Center, 2004) and averaged daily snow depth estimates within each study area. Daily minimum temperature values were obtained via remote sensing estimates from the PRISM Climate Group (2016) at the centroid of each study area. A daily winter severity index was calculated by first assigning each day one point if minimum temperature was <−17.8°C and one point if snow depth was >38.1 cm (Delgiudice et al., 2002). From this, a cumulative winter severity index was calculated by summing daily values for each winter beginning 1 November.

Snow‐free days were defined as days from 1 February to 31 May when mean snow depth was <7 cm, a depth that has been linked to behavioral and forage transitions for spring deer (Beier & McCullough, 1990). We predicted that deer mortality risk would respond gradually to snow‐free conditions during spring because mass gains for white‐tailed deer during spring are gradual (Delgiudice et al., 1992). Therefore, the number of snow‐free days was summed daily into cumulative snow‐free days from 1 February within each winter, which were then averaged within each weekly survival interval as a time‐varying covariate to reflect a possible cumulative effect in the relationship between deer mortality risk and spring snowmelt.

### 2.4 Survival analysis

Factors were assessed for influence on adult female deer weekly survival from 1 February to 31 May using Cox proportional hazards mixed‐effects models in the package coxme (Therneau, 2016) for program R (R Core Team, 2016). Because deer captured using Clover traps and rocket nets can experience capture myopathy‐related mortality (Beringer, Hansen, Wilding, Fisher, & Sheriff, 1996), deer were not included in survival models until 2 weeks postcapture. As yearling deer captured in this study had different patterns in body mass and pregnancy rates than older deer (Duquette, Belant, Beyer, & Jr, & Svoboda, N. J., 2012), survival analysis was limited to deer >2.5 years old.

Biological covariates of deer mortality risk included age (years) and body mass. Adult female survival was expected to follow a parabolic trend peaking at 5–6 years of age before declining (Delgiudice et al., 2006) and so it was modeled age as a polynomial quadratic term. Because deer body mass declined with capture date (see Results section for time–mass regression output), slope estimates from linear regressions of adult female body mass by capture date for each year were used to standardize body mass to 1 February (Festa‐Bianchet & Jorgenson, 1998).

Time‐varying covariates (Therneau, Crowson, & Atkinson, 2016) were included for cumulative winter severity index, cumulative snowmelt days, and snow depth, estimated for each week within years by averaging daily values over each weekly survival interval. A staggered entry design was used to account for varying capture dates of deer (Pollock, Winterstein, Bunck, & Curtis, 1989).

Low‐snowfall or mid‐snowfall study area were included as a random effect (i.e., frailty term) in all models to account for variation in predator populations, land cover, and other factors that may influence deer mortality risk among study areas (Pankratz, de Andrade, & Therneau, 2005). Multicollinearity among covariates was tested for using Spearman's rank correlation tests and considered any covariates with |*r*| < 0.7 suitable for inclusion in the same model (Dormann et al., 2013). Although Cox proportional hazards models have fewer assumptions than parametric survival models, an important assumption of the Cox model is that the baseline hazard ratio for each covariate remains constant over time. Violations of this assumption were assessed by testing for a significant (*p* < 0.05) interaction between weekly time period and each predictor covariate (Bellera et al., 2010; Fox & Weisberg, 2011). The final candidate model set included 24 candidate models with noncollinear covariate combinations of 6 factors: age, body mass, body mass:time interaction, weekly average snow depth, cumulative winter severity index, and cumulative snow‐free days. Because our goal was to explore the relative predictive value of model covariates on weekly deer survival, all candidate models were evaluated using backward stepwise model ranking based on Akaike's information criterion adjusted for small samples, where candidate models <2 ∆AIC_c_ of the top‐ranked model were considered plausible (Burnham & Anderson, 2002; Symonds & Moussalli, 2011).

## 3 RESULTS

One hundred and fifty pregnant adult female deer (>2.5 years old) were captured representing 1,784 deer‐weeks of monitoring during February–May. This included 147 unique individuals and 3 individuals that were recaptured and included in 2 years of monitoring. Median date of capture was 10 February (interquartile range = 21 January–25 February). Estimated age of captured deer ranged from 2.5 to 16.5 years (median = 6.5, interquartile range = 3.5–8.5). After standardization to 1 February, annual mean body mass of captured adult female deer ranged from 56.0 to 65.0 kg, and mean body mass within the mid‐snowfall study area (64.0 kg, *SD* = 5.8) was greater than the low‐snowfall study area (57.9 kg, *SD* = 7.2; *t*(116) = 5.57, *p* ≤ 0.001; Table 1). Among all capture years pooled, regression coefficient for body mass loss through the capture period was −0.88 kg/week (*n = *150, *r* ^2^ = 0.13, *p* < 0.001). Pooled across all years, mean weekly mortality rate during February–May (2.1%) was 3.5 times greater than mean weekly mortality during June–January (0.6%; obtained from the same study animals monitored year‐round every 2 weeks, but not included in mortality risk models).

**Table 1:** Summary of captured sample and survival covariates for radio‐collared adult female white‐tailed deer, Upper Peninsula of Michigan, USA, 1 February–31 May, 2009–2015

Winter	Study area	*N*	Survival	Mean body mass (*SD*)^a^	Cumulative winter severity^b^	Cumulative snow‐free days^b^
2009	Low‐snowfall	25	0.89	55.9 (6.1)	60	74
2010	Low‐snowfall	20	0.72	61.3(5.2)	11	81
2011	Low‐snowfall	18	0.74	56.8 (9.3)	15	74
2013	Mid‐snowfall	37	0.72	64.0 (5.6)	108	33
2014	Mid‐snowfall	27	0.22	65.0 (5.6)	167	38
2015	Mid‐snowfall	23	0.83	63.1 (6.5)	145	48

a Body mass adjusted to 1 February using regression by capture date.

b Cumulative values reflect values at the end of the monitoring period (31 May).

**Figure 3:**
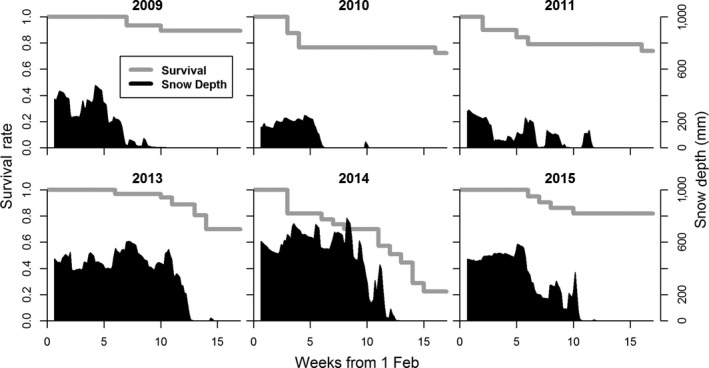
Weekly Kaplan–Meier survival estimates for 150 adult female white‐tailed deer (gray lines) and daily snow depth (shaded areas) from 1 February to 31 May, Upper Peninsula of Michigan, USA, within a low‐snowfall (2009–2011) study area and a mid‐snowfall (2013–2015) study area

Annual cumulative winter severity index values ranged from 11 to 167 (mean = 84.3, *SD* = 66.1), and annual cumulative snowmelt days ranged from 33 to 81 days (mean = 58, *SD* = 20; Table 1). Annual February–May survival estimates ranged from 0.22 to 0.89 (mean = 0.69, *SD* = 0.24). Weekly mortality rates were generally highest near the timing of snowmelt (Figure 3). We observed 44 mortality events, which we attributed to predation (*n* = 31), malnutrition (*n* = 8), drowning (*n* = 1), and unknown cause (*n* = 4; Table 2). Coyote (*n* = 12) and wolf (*n* = 11) were the most common predators of adult female deer, followed by unidentified predator (*n* = 6) and bobcat (*n* = 2). Of the 8 malnutrition mortalities, 6 occurred during the winter with greatest winter severity (2014; WSI = 167). No deer were censored due to radio‐collared failure or other reasons during the study interval.

**Table 2:** Known fates of radio‐collared adult female white‐tailed deer, Upper Peninsula of Michigan, USA, 1 February–31 May, 2009–2015

Winter	*n*	Predation				Malnutrition	Drowned	Unknown	Survived
		Bobcat	Coyote	Wolf	Unidentified				
2009	25	0	2	0	0	0	0	0	23
2010	20	0	1	1	1	0	0	0	17
2011	18	1	0	1	0	1	0	1	14
2013	39	1	5	2	1	1	0	1	28
2014	27	0	4	4	3	6	1	2	7
2015	24	0	0	3	1	0	0	0	20
Total	150	2	12	11	6	8	1	4	106

Snow depth and cumulative snow‐free days were collinear (*r* = 0.92) and were not included in the same model. Consequently, we did not have a single global model with all covariates included but rather two parallel sets of nested models. All other covariates had a correlation of |*r*| < 0.7. After accounting for multicollinear covariates and time interactions, we compared 24 candidate models (Table 3). The best‐supported model included body mass, cumulative winter severity index, and cumulative snow‐free days, but excluding body mass from this model also resulted in a plausible model (∆AICc = 0.994; Table 3). Within the best‐supported model, mortality risk increased 1.9% (95% CI = 0.8%–3.1%) with each unit increase in cumulative winter severity index, decreased 8.1% (95% CI = 3.9%–13.2%) with each cumulative snow‐free day, and decreased 4.3% (95% CI = 0%–8.8%) with each 1 kg increase in body mass. Scaled and centered covariate estimates of the best‐supported model suggested that deer mortality risk was most sensitive to cumulative snow‐free days (coeff. = −1.96, SE = 0.53), followed by cumulative winter severity (coeff. = 1.00, SE = 0.31) and body mass (coeff. = −0.32, SE = 0.18; Table 4).

**Table 3:** Weekly mortality risk effect estimates of covariates (data not transformed) and model selection results using Akaike's information criterion adjusted for small sample sizes (AICc) for Cox proportional hazards generalized linear mixed models estimating weekly mortality risk of radio‐collared adult female white‐tailed deer, Upper Peninsula of Michigan, USA, 1 February–31 May, 2009–2015

Covariate						AICc	∆AIC_c_
Age	Age^2^	Mass	Snow depth	SFD^a^	WSI^b^		
		−0.044		−0.085	0.0192	383.308	0.000
				−0.083	0.0187	384.302	0.994
−0.039	0.007	−0.048		−0.082	0.0197	385.718	2.410
−0.159	0.014			−0.083	0.0198	386.683	3.376
		−0.045		−0.068		391.930	8.622
				−0.069		393.441	10.133
0.056	0.000	−0.050		−0.071		394.351	11.043
		−0.043			0.0163	395.513	12.205
−0.096	0.010			−0.063		396.367	13.059
					0.0140	396.717	13.410
−0.012	0.007	−0.049			0.0182	396.737	13.429
		−0.040	−0.001		0.0168	397.254	13.947
			−0.001		0.0150	398.066	14.759
−0.122	0.013				0.0158	398.420	15.113
−0.009	0.006	−0.047	−0.001		0.0187	398.483	15.175
−0.106	0.012		−0.001		0.0168	399.770	16.462
		−0.034				407.367	24.059
						407.660	24.353
		−0.034	−0.001			408.496	25.189
			−0.001			408.681	25.373
0.072	−0.001	−0.043				409.754	26.446
0.073	−0.001	−0.043	−0.001			410.678	27.371
−0.030	0.005					410.767	27.459
−0.025	0.005		−0.001			411.620	28.312

Note

SFD represents weekly cumulative snow‐free days, and WSI represents weekly cumulative winter severity index. All models included study area as a random effect.

a Cumulative snow‐free days from 1 November to 31 May.

b Cumulative winter severity index from 1 November to 31 May (Delgiudice et al., 2002).

**Table 4:** Estimates of scaled and centered covariates from the top‐ranked Cox proportional hazards generalized linear mixed model for weekly mortality risk of radio‐collared adult female white‐tailed deer, Upper Peninsula of Michigan, USA, 2009–2015.

Covariate	Coeff.	SE	*Z*	*p*
Body mass	−0.316	0.176	−1.80	0.072
Cumulative winter severity index	1.008	0.308	3.28	0.001
Cumulative snow‐free days	−1.961	0.534	−3.67	<0.001

Note

Model included study area as a random effect.

## 4 DISCUSSION

The influence of winter weather on white‐tailed deer winter mortality risk which we observed is consistent with other studies of deer survival in northern climates (e.g., Delgiudice et al., 2002; Dumont et al., 2000; Nelson & Mech, 1986), and generally supports the hypothesis that winter weather conditions are the primary factor limiting the northern distribution of white‐tailed deer in North America (Dawe, Bayne, & Boutin, 2014; Kennedy‐Slaney, Bowman, Walpole, & Pond, 2018). Our results indicated that the most critical period of winter deer mortality risk is late winter–early spring (April and May) when snowmelt occurs, but mortality risk varied widely among years depending on weather conditions. However, by assuming an immediate reduction in mortality risk following spring snowmelt our model may estimate the effects of snowmelt on mortality risk to act somewhat faster than realistic. For example, while our model indicated mortality risk to be greatest immediately before snowmelt, observed mortality rates during 2013 and 2014 (the two years with latest snowmelt) remained high for 1–2 weeks following snowmelt (Figure 3). This could indicate a lag effect of snow conditions on deer mortality risk which our model did not account for. Such a lag effect may be expected because following snowmelt, deer physical condition is likely at an annual nadir, and nutritional recovery from winter likely takes several weeks to begin (Delgiudice et al., 1992). Additionally, because we only modeled deer survival during February–May it is possible that factors influencing mortality during early winter differ from those in our mortality risk models. For example, it is possible that some deer entering winter in exceptionally poor condition may not have survived to 1 February.

Our best‐supported model included a survival advantage for deer with greater body mass, but a closely competing model without body mass suggests that the effect of mass was weak, if present. A survival advantage for individuals with greater body mass has been noted in other ungulate populations with winter nutritional deficits such as red deer (*Cervus elaphus* [L. 1758]; Lioson, Langvatn, & Solberg, 1999). There are several plausible explanations for why body mass would be positively correlated with winter deer survival in our study. First, in northern white‐tailed deer there are pronounced seasonal changes in body mass following nutritional gains and losses (Delgiudice et al., 1992). Within individuals, changes in mass are a good index of condition because ungulate body mass is correlated with condition because increasing fat stores adds body mass to individuals (Stephenson, Hundertmark, Schwartz, & Ballenberghe, 1998), and ungulates mobilize both fat and lean body tissue according to their condition at the onset of winter to meet the energetic demands of survival and pregnancy (Monteith et al., 2013). However, among individuals there are differences in skeletal size and lean body mass, unrelated to body fat reserves, which we did not account for by measuring body mass alone. Certainly, this adds unaccounted for variation to the effect of body mass on mortality risk in our study. Second, larger homeothermic vertebrates with greater lean body mass can carry more energy reserves relative to metabolic weight and endure longer periods of fasting (Boyce, 1979; Lindstedt & Boyce, 1985). Hence, it is possible that a larger skeletal frame provides a survival advantage for northern ungulates. By only measuring deer body mass and not actual fat reserves, we cannot reach conclusions about which of these processes may have been important in our study. However, the considerably larger body mass of deer in the mid‐snowfall study area does support the result of our mortality risk models, indicating a selective advantage for larger deer during longer winters.

Our results suggest that deer mortality in the Upper Peninsula may be similarly or more sensitive to the timing of spring snowmelt than temperatures or snow depth throughout early–mid‐winter. Previous research suggests that northern deer may lose body mass during winter at a similar rate during mild or severe winters (Giroux, Dussault, Tremblay, & Cote, 2016), which along with our results could indicate that the difference between a mild and severe winter from a deer energetic perspective is more determined by seasonal snow cover duration than by short‐term (within‐week) snow depth or temperature conditions. The number of snow‐free days in February–May is likely correlated with WSI on an annual scale, but two winters can have similar WSIs with notable differences in spring snowmelt. For example, winter 2012–2013 (winter severity index = 108; 33 snow‐free days) had a lesser total winter severity index but 15 more days of snow cover during February–May than winter 2014–2015 (winter severity index = 145; 48 snow‐free days).

If the immediate physical effects of deep snow influenced deer mortality risk by impeding the ability to escape predators, snow depth should be positively correlated with deer mortality risk. In our study, weekly snow depth did not explain variation in weekly deer mortality risk, but other studies have found contrasting results. In Minnesota, wolf predation rates on yearling and adult white‐tailed deer were the greatest during months with the deepest snow (Nelson & Mech, 1986), and daily wolf kill rates of deer in a high snowfall area of Michigan were highly correlated with snow depth in a previous study (Vucetich et al., 2012). These studies suggest that the immediate effects of deep snow can increase predation on deer in some circumstances, but our results suggest that the gradual nutritional decline throughout winter was the primary mechanism influencing mortality risk during our study. A similar nutritional influence on white‐tailed deer mortality was observed in South Dakota, where poor winter range conditions resulted in April–June adult female mortality rates exceeding 20% in 3 of 4 years (DePerno et al., 2000). Among nearby white‐tailed deer studies using the same Winter Severity Index as ours, a WSI of 167 as we observed in winter 2013–20114 is associated with poor deer population performance; in Ontario, Canada, Dawe et al. (2014) predicted a <10% probability of deer populations occurring in areas with an average WSI ≥ 167. In Minnesota, USA, Delgiudice et al. (2002) reported one winter with WSI > 167 (WSI = 199), with a 46% mortality rate for adult female deer. This suggests that cumulative winter snow and temperature conditions experienced by deer in our study during the winter of 2013–2014 were at the upper end of white‐tailed deer tolerance, which is supported by the exceptionally high deer mortality rate we observed.

There are several possible explanations for why deer mortality risk in our study was strongly influenced by snow cover during late winter. First, pregnant female deer have a 45% increase in metabolic demands entering the third trimester of pregnancy (Pekins, Smith, & Mautz, 1998), which could result in a greater energy deficit for pregnant females during April and May even if dietary quality is similar during early winter. We confirmed all deer in our study as pregnant within the winter of their survival analysis; however, we did not know whether individuals had birthed or successfully weaned fawns within the preceding year, which can have a strong influence on ungulate condition in subsequent fall/winter (e.g., Simard, Huot, Bellefeuille, & Côté, 2014). Likely as a result of pregnancy, declining forage, and cumulative energetic expenditure since the onset of winter, adult female northern deer are at an annual nutritional nadir during May (Delgiudice et al., 1992). Finally, crusted snow conditions during spring facilitate deer predation by wolves and coyotes because of heavier foot‐loading in deer (Telfer & Kelsall, 1984; Vucetich et al., 2012). With many deer in poor physical condition and snow conditions that favor predator movement, among‐year differences of several weeks in the timing of spring snowmelt could have a substantial effect on deer vulnerability to predation or malnutrition mortality.

A decrease in deer mortality risk following snowmelt may be the result of several processes. First, deer foraging during deep snow conditions is mostly limited to food available along established trails, where preferred browse species become depleted throughout winter (DelGiudice, Sampson, & Giudice, 2013). Hobbs (1989) predicted that the energetic losses due to reduced forage intake and locomotion in deep snow were 5.4 times greater than losses due to increased thermoregulatory expenses in cold temperatures for mule deer (*Odocoileous hemonius* [Rafinesque 1817]). Conditions of little or no snow depth facilitate movement and allow deer access to additional woody browse and ground forages. However, even with a positive energy budget, spring body mass gain by deer is a gradual process of weeks or months (Delgiudice et al., 1992). Consequently, deer may remain in relatively poor condition for several weeks following snowmelt before making a nutritional recovery. In addition, many deer in Michigan's Upper Peninsula undergo spring migration of up to 80 kilometers to traditional summer ranges shortly after snowmelt (Van Deelen, Campa, Hamady, & Haufler, 1998). Possibly, the return of deer to more widely dispersed summer ranges from concentrated winter ranges could reduce predation risk by reducing predator encounter rates.

Overall, our study supports the nutritional integration model applied to northern white‐tailed deer, which adds further support to the already substantial evidence that weather conditions, through nutritional effects, are the primary mediator of northern ungulate population dynamics (Parker et al., 2009; Post & Stenseth, 1999). However, the specific environmental and individual conditions important to deer mortality risk in our study may not apply broadly to other northern ungulates. For example, while it appears that duration of snow cover and the timing of spring snowmelt were critical factors for deer in our study, reindeer (*Rangifor tarandus tarandus* [L. 1758]) populations tend to be most sensitive to winter icing conditions (Helle & Kojola, 2008; Hansen, Aanes, Herfindal, Kohler, & Sæther, 2011), and moose (*Alces alces* [L. 1758]) population growth on Isle Royale National Park, USA, showed little or no response to winter precipitation, although snow conditions on the ground may still be important (Vucetich & Peterson 2003). Additionally, within a single species the types of winter weather conditions that result in nutritional stress population declines may differ considerably among regions (e.g., reindeer; Tyler, 2010). Finally, varying degrees of predation pressure among ungulate populations may interact with winter weather and nutrition, as is the case with adult female elk (Brodie et al., 2013) and mule deer (Forrester & Wittmer, 2012) in western North America. Consequently, conditions leading to nutritionally mediated survival bottlenecks (if present) are likely to vary considerably among populations of northern ungulates. Identifying factors that result in ungulate survival bottlenecks at a local level may be very useful for management decisions.

## 5 CONCLUSIONS

We based our predictions on the hypothesis that deer generally maintain a negative energy balance during winter at northern latitudes and survival is dependent on conserving energetic stores until spring snowmelt. A positive relationship between cumulative winter severity index and mortality risk suggests that winters with deep snow and cold temperatures accelerate the decline of deer condition. A plausible, albeit weak, negative relationship between body mass and mortality risk suggests that larger deer are less susceptible to nutritional decline during late winter. Finally, a negative relationship between snow‐free days and mortality risk suggests that late‐persisting deep snow conditions at the end of winter strongly increase mortality risk. Taken together, these conclusions suggest that deer in this population have a relatively low risk of mortality even under conditions of deep snow, as long as adequate nutritional reserves remain. However, once nutritional reserves are depleted, female deer of all age classes can experience this increased mortality risk, resulting in a survival bottleneck. Overall, this supports the critical role of fat reserves for white‐tailed deer winter survival as suggested by Mautz (1978), and more broadly supports the nutritional integration model of northern ungulate ecology suggested by Parker et al. (2009). In future studies of northern ungulates with a negative energy budget during periods of snow cover, considering snow‐free days during late winter or a similar measure of spring snowmelt timing may improve model accuracy.

## AUTHOR CONTRIBUTIONS

TK, JB, DB, and BS conceived the ideas and designed methodology. TK and JD collected the data. TK and JB analyzed the data. TK and JB led the writing of the manuscript. All authors contributed critically to the drafts and gave final approval for publication.

## Data Availability

Data used in this manuscript have been archived within the Dryad Digital Repository (available at https://doi.org/10.5061/dryad.djh9w0vwb).

## References

[ece36026-bib-0001] Ashmole, N. P. (1963). The regulation of numbers of tropical oceanic birds. IBIS, 103(3), 458–473. 10.1111/j.1474-919X.1963.tb06766.x

[ece36026-bib-0002] Beier, P. , & McCullough, D. R. (1990). Factors influencing white‐tailed deer activity patterns and habitat use. Wildlife Monographs, 109, 3–51.

[ece36026-bib-0003] Bellera, C. A. , MacGrogan, G. , Debled, M. , de Lara, C. T. , Brouste, V. , & Mathoulin‐Pelissier, S. (2010). Variables with time‐varying effects and the Cox model: Some statistical concepts illustrated with a prognostic factor study in breast cancer. BMC Medical Research Methodology, 10(1), 20 10.1186/1471-2288-10-20 20233435 PMC2846954

[ece36026-bib-0004] Beringer, J. , Hansen, L. P. , Wilding, W. , Fisher, J. , & Sheriff, S. L. (1996). Factors affecting capture myopathy in white‐tailed deer. Journal of Wildlife Management, 60(2), 373–380. 10.2307/3802238

[ece36026-bib-0005] Boyce, M. S. (1979). Seasonality and patterns of natural selection for life histories. The American Naturalist, 114, 569–583. 10.1086/283503

[ece36026-bib-0006] Brodie, J. , Johnson, H. , Mitchell, M. , Zager, P. , Proffitt, K. , Hebblewhite, M. , … White, P. J. (2013). Relative influence of human harvest, carnivores, and weather on adult female elk survival across western North America. Journal of Applied Ecology, 50(2), 295–305. 10.1111/1365-2664.12044

[ece36026-bib-0007] Burnham, K. P. , & Anderson, D. E. (2002). Model selection and multimodel inference: A practical information‐theoretic approach (2nd ed.). New York, NY: Springer‐Verlag.

[ece36026-bib-0008] Case, D. J. , & McCullough, D. R. (1987). The white‐tailed deer of North Manitou Island. Hilgardia, 55(9), 1–57. 10.3733/hilg.v55n09p057

[ece36026-bib-0009] Chadwick, S. B. (2002). Automating a winter severity index for Michigan Wildlife (Report 3375). Lansing, MI: Michigan Department of Natural Resources Wildlife.

[ece36026-bib-0010] Clover, M. R. (1956). Single‐gate deer trap. California Fish and Game, 42, 199–201.

[ece36026-bib-0011] Clutton‐Brock, T. H. , Price, O. F. , Albon, S. D. , & Jewell, P. A. (1991). Persistent instability and population regulation in Soay Sheep. Journal of Animal Ecology, 60(2), 593–608. 10.2307/5300

[ece36026-bib-0012] Dawe, K. L. , Bayne, E. M. , & Boutin, S. (2014). Influence of climate and human land use on the distribution of white‐tailed deer (*Odocoileus virginianus*) in the western boreal forest. Canadian Journal of Zoology, 92(4), 353–363. 10.1139/cjz-2013-0262

[ece36026-bib-0013] DelGiudice, G. D. , Fieberg, J. , Riggs, M. R. , Carstenson Powell, M. , & Pan, W. (2006). A long‐term age‐specific survival analysis of female white‐tailed deer. Journal of Wildlife Management, 70(6), 1556–1568. 10.2193/0022-541X(2006)70[1556:ALASAO]2.0.CO;2

[ece36026-bib-0014] DelGiudice, G. D. , Mech, L. D. , Kunkel, K. E. , Gese, E. M. , & Seal, U. S. (1992). Seasonal patterns of weight, hematology, and serum characteristics of free‐ranging female white‐tailed deer in Minnesota. Canadian Journal of Zoology, 70(5), 974–983. 10.1139/z92-139

[ece36026-bib-0015] DelGiudice, G. D. , Riggs, M. R. , Joly, P. , & Pan, W. (2002). Winter severity, survival, and cause‐specific mortality of female white‐tailed deer in north‐central Minnesota. Journal of Wildlife Management, 66(3), 698–717. 10.2307/3803136

[ece36026-bib-0016] DelGiudice, G. D. , Sampson, B. A. , & Giudice, J. H. (2013). A long‐term assessment of the effect of winter severity on the food habits of white‐tailed deer. Journal of Wildlife Management, 77(8), 1664–1675. 10.1002/jwmg.616

[ece36026-bib-0017] DePerno, C. S. , Jenks, J. A. , Griffin, S. L. , & Rice, L. A. (2000). Female survival rates in a declining white‐tailed deer population. Wildlife Society Bulletin, 28(4), 1030–1037.

[ece36026-bib-0018] Doepker, R. V. , Beyer, D. E. , & Donovan, M. (1995). Deer population trends in Michigan's Upper Peninsula (Report 3254). Lansing, MI: Michigan Department of Natural Resources Wildlife Division.

[ece36026-bib-0019] Dormann, C. F. , Elith, J. , Bacher, S. , Buchmann, C. , Carl, G. , Carré, G. , … Lautenbach, S. (2013). Collinearity: A review of methods to deal with it and a simulation study evaluating their performance. Ecography, 36(1), 27–46. 10.1111/j.1600-0587.2012.07348.x

[ece36026-bib-0020] Dumont, A. , Crête, M. , Ouellet, J. P. , Huot, J. , & Lamoureux, J. (2000). Population dynamics of northern white‐tailed deer during mild winters: Evidence of regulation by food competition. Canadian Journal of Zoology, 78(5), 764–776. 10.1139/z99-264

[ece36026-bib-0021] Dumont, A. , Ouellet, J. P. , Crête, M. , & Huot, J. (2005). Winter foraging strategy of white‐tailed deer at the northern limit of its range. Ecoscience, 12(4), 476–484. 10.2980/i1195-6860-12-4-476.1

[ece36026-bib-0022] Duquette, J. F. , Belant, J. L. , Beyer, D. E., Jr. , & Svoboda, N. J. (2012). Comparison of pregnancy detection in live white‐tailed deer. Wildlife Society Bulletin, 36(1), 115–118. 10.1002/wsb.115

[ece36026-bib-0023] Duquette, J. F. , Belant, J. L. , Beyer, D. E. Jr , & Svoboda, N. J. (2013). Body condition and dosage effects on ketamine‐xylazine immobilization of female white‐tailed deer. Wildlife Society Bulletin, 37(1), 162–167. 10.1002/wsb.233

[ece36026-bib-0024] Duquette, J. F. , Belant, J. L. , Svoboda, N. J. , Beyer, D. E. , Lederle, P. E. , & Roca, A. L. (2014). Effects of Maternal Nutrition, Resource Use and Multi-Predator Risk on Neonatal White-Tailed Deer Survival. PLoS ONE, 9(6), e100841 10.1371/journal.pone.0100841 24968318 PMC4072703

[ece36026-bib-0025] Ericsson, G. , & Wallin, K. (2001). Age‐specific moose (*Alces alces*) mortality in a predator‐free environment: Evidence for senescence in females. Ecoscience, 8(2), 157–163.

[ece36026-bib-0026] Festa‐Bianchet, M. , & Jorgenson, J. T. (1998). Selfish mothers: Reproductive expenditure and resource availability in bighorn ewes. Behavioral Ecology, 9(2), 144–150. 10.1093/beheco/9.2.144

[ece36026-bib-0027] Forrester, T. D. , & Wittmer, H. U. (2012). A review of the population dynamics of mule deer and black‐tailed deer *Odocoileus hemionus* in North America. Mammal Review, 43(4), 292–308. 10.1111/mam.12002

[ece36026-bib-0028] Fox, J. , & Weisberg, S. (2011). Cox proportional‐hazards regression for survival data in R (appendix): An R companion to applied regression (2nd ed). Thousand Oaks, CA: Sage.

[ece36026-bib-0029] Fry, J. , Xian, G. , Jin, S. , Dewitz, J. , Homer, C. , Yang, L. , … Wickam, J. (2011). Completion of the 2006 National Land Cover Database for the conterminous United States. Photogrammetric Engineering and Remote Sensing, 77(9), 858–864.

[ece36026-bib-0030] Gaillard, J. M. , Festa‐Bianchet, M. , & Yoccoz, N. G. (1998). Population dynamics of large herbivores: Variable recruitment with constant adult survival. Trends in Ecology and Evolution, 13(2), 58–63. 10.1016/S0169-5347(97)01237-8 21238201

[ece36026-bib-0031] Gaillard, J. M. , & Yoccoz, N. G. (2003). Temporal variation in survival of mammals: A case of environmental canalization? Ecology, 84(12), 3294–3306. 10.1890/02-0409

[ece36026-bib-0032] Gilbert, F. F. (1966). Aging white‐tailed deer by annuli in the cementum of the first incisor. Journal of Wildlife Management, 30(1), 200–202. 10.2307/3797906

[ece36026-bib-0033] Giroux, M. A. , Dussault, C. , Tremblay, J. P. , & Cote, S. D. (2016). Winter severity modulates the benefits of using a habitat temporally uncoupled from browsing. Ecosphere, 7, e01432 10.1002/ecs2.1432

[ece36026-bib-0034] Hansen, B. B. , Aanes, R. , Herfindal, I. , Kohler, J. , & Sæther, B. E. (2011). Climate, icing, and wild arctic reindeer: Past relationships and future prospects. Ecology, 92(10), 1917–1923. 10.1890/11-0095.1 22073783

[ece36026-bib-0035] Helle, T. , & Kojola, I. (2008). Demographics in an alpine reindeer herd: effects of density and winter weather. Ecography, 31(2), 221–230. 10.1111/j.0906-7590.2008.4912.x

[ece36026-bib-0036] Hobbs, N. T. (1989). Linking energy balance to survival in mule deer: Development and test of a simulation model. Wildlife Monographs, 101, 3–39.

[ece36026-bib-0037] Kennedy‐Slaney, L. , Bowman, J. , Walpole, A. A. , & Pond, B. A. (2018). Northward bound: The distribution of white‐tailed deer in Ontario under a changing climate. Wildlife Research, 45(3), 220–228. 10.1071/WR17106

[ece36026-bib-0038] Kreeger, T. J. , Arnemo, J. M. , & Raath, J. P. (2002). Handbook of wildlife chemical immobilization (International ed.). Fort Collins, CO: Wildlife Pharmaceuticals.

[ece36026-bib-0039] Leckenby, D. A. , & Adams, A. W. (1986). A weather severity index on a mule deer winter range. Journal or Range Management, 39, 244–248. 10.2307/3899059

[ece36026-bib-0040] Leopold, A. , Sowls, L. K. , & Spencer, D. L. (1947). A survey of over‐populated deer ranges in the United States. Journal of Wildlife Management, 11(2), 162–177. 10.2307/3795561

[ece36026-bib-0041] Lindstedt, S. L. , & Boyce, M. S. (1985). Seasonality, fasting endurance, and body size in mammals. The American Naturalist, 125(6), 873–878. 10.1086/284385

[ece36026-bib-0042] Loison, A. , Festa‐Bianchet, M. , Gaillard, J. M. , Jorgenson, J. T. , & Jullien, J. M. (1999). Age‐specific survival in five populations of ungulates: Evidence of senescence. Ecology, 80(8), 2539–2554. 10.1890/0012-9658(1999)080[2539:ASSIFP]2.0.CO;2

[ece36026-bib-0043] Loison, A. , Langvatn, R. , & Solberg, E. J. (1999). Body mass and winter mortality in red deer calves: Disentangling sex and climate effects. Ecography, 22(1), 20–30. 10.1111/j.1600-0587.1999.tb00451.x

[ece36026-bib-0044] Mautz, W. W. (1978). Sledding on a bushy hillside: The fat cycle in deer. Wildlife Society Bulletin, 6(2), 88–90.

[ece36026-bib-0045] McCullough, D. R. (1979). The George Reserve deer herd: Population Ecology of a K‐selected species. Ann Arbor, MI: University of Michigan Press.

[ece36026-bib-0046] Mech, L. D. (2008). Precision of descriptors for percent marrow fat content for white‐tailed deer, *Odocoileus viriginianus* . Canadian Field Naturalist, 122(3), 273 10.22621/cfn.v122i3.615

[ece36026-bib-0047] Mech, L. D. , Frenzel, D. , & Karns, P. D. (1971). The effect of snow conditions on the vulnerability of white‐tailed deer to wolf predation In Mech L. D. & Frenzel L. D. Jr. (Eds.), Ecological studies of the timber wolf in northeastern Minnesota (Research Paper NC‐52, pp. 51–59). St. Paul, MA: U.S. Forest Service.

[ece36026-bib-0048] Mech, L. D. , & Peterson, R. O. (2003). Wolf‐prey relations In Mech L. D., & Boitani L. (Eds.), Wolves: Behavior, ecology, and conservation (pp. 131–215). Chicago, IL: University of Chicago Press.

[ece36026-bib-0049] Mech, L. D. , Smith, D. W. , Murphy, K. M. , & MacNulty, D. R. (2001). Winter severity and wolf predation on a formerly wolf‐free elk herd. Journal of Wildlife Management, 65(4), 998–1003. 10.2307/3803048

[ece36026-bib-0050] Metz, M. C. , Smith, D. W. , Vucetich, J. A. , Stahler, D. R. , & Peterson, R. O. (2012). Seasonal patterns of predation for gray wolves in the multi‐prey system of Yellowstone National Park. Journal of Animal Ecology, 81(3), 553–563. 10.2193/0022-541X(2004)068[0153:WPSAEO]2.0.CO;2 22260633

[ece36026-bib-0051] Michigan Department of Natural Resources . (2010). Michigan deer management plan (Report 3512). Lansing, MI: Michigan Department of Natural Resources and Environment Wildlife Division Retrieved from http://www.michigan.gov/dnr/0,4570,7-153-10363_41153%2013-,00.html

[ece36026-bib-0052] Michigan Department of Natural Resources . (2015). Michigan wolf management plan (Report 3604). Lansing, MI: Michigan Department of Natural Resources and Environment Wildlife Division Retrieved from https://www.michigan.gov/documents/dnr/Draft_Wolf_Management_Plan_030708_227742_7.pdf

[ece36026-bib-0053] Michigan State Climatology Office . (2013a). Climate normals and extremes by month. Escanaba Station 2626 Retrieved from http://climate.geo.msu.edu/Stations/2626/monthly.pdf

[ece36026-bib-0054] Michigan State Climatology Office . (2013b). Climate normals and extremes by month. Crystal Falls Station 1922 Retrieved from http://climate.geo.msu.edu/Stations/2626/monthly.pdf

[ece36026-bib-0055] Monteith, K. L. , Stephenson, T. R. , Bleich, V. C. , Conner, M. M. , Pierce, B. M. , & Bowyer, R. T. (2013). Risk‐sensitive allocation in seasonal dynamics of fat and protein reserves in a long‐lived mammal. Journal of Animal Ecology, 82(2), 377–388. 10.1111/1365-2656.12016 23379674

[ece36026-bib-0056] National Operational Hydrologic Remote Sensing Center . (2004). Snow data assimilation system (SNODAS) data products at NSIDC, version 1. Daily snow depth. Boulder, CO: Author 10.7265/N5TB14TC

[ece36026-bib-0057] Nelson, M. E. (1995). Winter range arrival and departure of white‐tailed deer in northeastern Minnesota. Canadian Journal of Zoology, 73(6), 1069–1076. 10.1139/z95-127

[ece36026-bib-0058] Nelson, M. E. (2002). The science ethics and philosophy of tooth extractions from live‐captured white‐tailed deer: a response to Festa‐Bianchet et al (2002). Wildlife Society Bulletin, 30(1), 284–288.

[ece36026-bib-0059] Nelson, M. E. , & Mech, L. D. (1986). Mortality of white‐tailed deer in northeastern Minnesota. Journal of Wildlife Management, 50(4), 691–698. 10.2307/3800983

[ece36026-bib-0060] Pankratz, V. S. , de Andrade, M. , & Therneau, T. M. (2005). Random‐effects Cox proportional hazards model: General variance components methods for time‐to‐event data. Genetic Epidemiology, 28(2), 97–109. 10.1002/gepi.20043 15532036

[ece36026-bib-0061] Parker, K. L. , Barboza, P. S. , & Gillingham, M. P. (2009). Nutrition integrates environmental responses of ungulates. Functional Ecology, 23(1), 57–69. 10.1111/j.1365-2435.2009.01528.x

[ece36026-bib-0062] Pekins, P. J. , Smith, K. S. , & Mautz, W. W. (1998). The energy cost of gestation in white‐tailed deer. Canadian Journal of Zoology, 76(6), 1091–1097. 10.1139/z98-032

[ece36026-bib-0063] Pollock, K. H. , Winterstein, S. R. , Bunck, C. M. , & Curtis, P. D. (1989). Survival analysis in telemetry studies: The staggered entry design. Journal of Wildlife Management, 53(1), 7–15. 10.2307/3801296

[ece36026-bib-0064] Post, E. , & Stenseth, N. C. (1999). Climatic variability, plant phenology, and northern ungulates. Ecology, 80(4), 1322–1339. 10.1890/0012-9658(1999)080[1322:CVPPAN]2.0.CO;2

[ece36026-bib-0065] PRISM Climate Group, Oregon State University . (2016). Daily minimum temperature datasets. Retrieved from http://www.prismclimate.org

[ece36026-bib-0066] R Core Team . (2016). R: A language and environment for statistical computing. Vienna, Austria: R Foundation for Statistical Computing Retrieved from http://www.R-project.org/

[ece36026-bib-0067] Simard, M. A. , Huot, J. , de Bellefeuille, S. , & Côté, S. D. (2014). Linking conception and weaning success with environmental variation and female body condition in a northern ungulate. Journal of Mammalogy, 95(2), 311–327. 10.1644/13-MAMM-A-036

[ece36026-bib-0068] Stephenson, T. R. , Hundertmark, K. J. , Schwartz, C. C. , & Ballenberghe, V. V. (1998). Predicting body fat and body mass in moose with ultrasonography. Canadian Journal of Zoology, 76(4), 717–722. 10.1139/z97-248

[ece36026-bib-0069] Symonds, R. E. , & Moussalli, A. (2011). A brief guide to model selection, multimodel inference, and model averaging in behavioral ecology using Akaike's information criterion. Behavioral Ecology and Sociobiology, 65(1), 13–21. 10.1007/s00265-010-1037-6

[ece36026-bib-0070] Telfer, E. S. , & Kelsall, J. P. (1984). Adaptation of some large North American mammals for survival in snow. Ecology, 65(6), 1828–1834. 10.2307/1937779

[ece36026-bib-0071] Therneau, T. (2016). coxme: Mixed effects Cox models. R package ver 2.2–7. https://cran.r-project.org/web/packages/coxme/index.html

[ece36026-bib-0072] Therneau, T. , Crowson, C. , & Atkinson, E. (2016). Using time dependent covariates and time dependent coefficients in the Cox model. Survival Vignettes. Retrieved from ftp://ftp.br.debian.org/CRAN/web/packages/survival/vignettes/timedep.pdf

[ece36026-bib-0073] Tyler, N. J. C. (2010). Climate, snow, ice, crashes, and declines in populations of reindeer and caribou (Rangifer tarandus L.). Ecological Monographs, 80(2), 197–219. 10.1890/09-1070.1

[ece36026-bib-0074] Van Deelen, T. R. , Campa, H. III , Hamady, M. , & Haufler, J. B. (1998). Migration and seasonal range dynamics of deer using adjacent deeryards in northern Michigan. Journal of Wildlife Management, 62(1), 205–213. 10.2307/3802280

[ece36026-bib-0075] Van Deelen, T. R. , Campa, H. III , Haufler, J. B. , & Thompson, P. D. (1997). Mortality patterns of white‐tailed deer in Michigan's Upper Peninsula. Journal of Wildlife Management, 61(3), 903–910. 10.2307/3802199

[ece36026-bib-0076] Verme, L. J. (1968). An index of winter weather severity for northern deer. Journal of Wildlife Management, 32(3), 566–574. 10.2307/3798937

[ece36026-bib-0077] Verme, L. J. (1977). Assessment of natal mortality in Upper Michigan deer. Journal of Wildlife Management, 41(4), 700–708. 10.2307/3799992

[ece36026-bib-0078] Vucetich, J. A. , Huntzinger, B. A. , Peterson, R. O. , Vucetich, L. M. , Hammill, J. H. , & Beyer, D. E. Jr (2012). Intra‐seasonal variation in wolf *Canis lupus* kill rates. Wildlife Biology, 18(3), 235–245. 10.2981/11-061

[ece36026-bib-0079] Wang, G. , Hobbs, N. T. , Twombly, S. , Boone, R. B. , Illius, A. W. , Gordon, I. J. , & Gross, J. E. (2009). Density dependence in northern ungulates: Interactions with predation and resources. Population Ecology, 51(1), 123–132. 10.1007/s10144-008-0095-3

[ece36026-bib-0080] Whitlaw, H. A. , Ballard, W. B. , Sabine, D. L. , Young, S. J. , Jenkins, R. A. , & Forbes, G. J. (1998). Survival and cause‐specific mortality rates of adult white‐tailed deer in New Brunswick. Journal of Wildlife Management, 62(4), 1335–1341. 10.2307/3801999

