# Supplementary material for: Influence of body mass and environmental conditions on winter mortality risk of a northern ungulate: Evidence for a late‐winter survival bottleneck
Source: Ecol Evol. 2020 Jan 21;10(3):1666–77. doi: 10.1002/ece3.6026 (PMC7029083; doi:10.1002/ece3.6026)
Supplement: Supplementary file 1 [file ECE3-10-1666-s001.docx]

**Table S1: Spearman rank correlations (**|*r*|)  **among covariates for Cox Proportional Hazard models of mortality risk for adult female white-tailed deer in the Upper Peninsula of Michigan, USA, Feb–May, 2009–2015.**

|  | Body Mass | Age | Snow Depth | Cumulative WSI | Cumulative Snow Free Days |
| --- | --- | --- | --- | --- | --- |
| Body Mass | 1 | - | - | - | - |
| Age | 0.17 | 1 | - | - | - |
| Snow Depth | 0.22 | -0.06 | 1 | - | - |
| Cumulative WSI | 0.28 | -0.15 | 0.24 | 1 | - |
| Cumulative Snow Free Days | -0.24 | 0.08 | -0.92 | -0.32 | 1 |
